# Case Report: A novel 1, 171 bp GCK promoter/exon 1 deletion in a Chinese pedigree: correcting a misdiagnosis of MODY10 based on an incidental INS variant

**DOI:** 10.3389/fendo.2026.1967659

**Published:** 2026-09-17

**Authors:** Jiahui Chen, Xingwu Zhang, Di Wu, Yanmei Sang, Xianze Wang

**Affiliations:** 1 Department of Endocrinology, Genetics, and Metabolism, Beijing Children’s Hospital, Capital Medical University, National Center for Children’s Health, Beijing, China; 2 School of Medicine, Tsinghua University, Beijing, China; 3 Peking Union Medical College Hospital, Beijing, China; 4 Department of General Surgery, Peking Union Medical College Hospital, Beijing, China; 5 State Key Laboratory of Complex Severe and Rare Diseases, Peking Union Medical College Hospital, Beijing, China

**Keywords:** continuous glucose monitoring, copy number variation, maturity-onset diabetes of the young, MODY10, MODY2, monogenic diabetes, variant misclassification

## Abstract

**Background:**

Maturity-onset diabetes of the young (MODY) comprises a heterogeneous group of autosomal dominant monogenic diabetes disorders; accurate molecular diagnosis is essential, as different subtypes require fundamentally distinct management strategies.

**Objective:**

In a Chinese pedigree initially misdiagnosed as MODY10 based on an incidental INS variant, we aimed to identify the true genetic etiology through comprehensive genetic analysis (including copy number variation [CNV] detection) and continuous glucose monitoring (CGM), and to illustrate the clinical consequences of rigorous variant interpretation.

**Methods:**

Detailed clinical evaluation, whole-exome sequencing (WES), whole-genome sequencing (WGS), multiplex ligation-dependent probe amplification (MLPA), segregation analysis, and CGM monitoring were performed across three generations of the pedigree.

**Results:**

We identified a 1, 171 bp GCK deletion (chr7:44, 228, 224-44, 229, 394 [GRCh37], encompassing the β-cell promoter, exon 1, and part of intron 1) that co-segregated with diabetes across three generations. This deletion removes the β-cell promoter while leaving the hepatic promoter intact, predicting selective loss of the pancreatic glucokinase isoform. The deletion was classified as pathogenic under ACMG/AMP criteria using the ClinGen Monogenic Diabetes Expert Panel specifications. Segregation analysis revealed that an INS variant (NM_000207.3:c.4G>A, p.Ala2Thr; rs535989053) did not co-segregate with diabetes: it was inherited from the euglycaemic maternal grandfather (fasting glucose 5.0 mmol/L, HbA1c 5.2%) and was absent in the proband. Under ACMG/AMP criteria, the INS variant meets criteria for a benign allele (BS1, BP2). The proband (isolated MODY2) exhibited a characteristic “plateau-like” stable mild hyperglycemia (coefficient of variation [CV] 14.35%). The elder brother, who carries both variants, showed a similar stable pattern (CV 13.41%). The mother, who also carries both variants, had been treated with insulin for ten years under the erroneous MODY10 diagnosis; upon discontinuation of insulin, her glycemic control remained stable, with CGM metrics comparable to those of her children.

**Conclusion:**

This case underscores the critical importance of CNV analysis and segregation studies in monogenic diabetes diagnostics. The identification of a large GCK deletion corrected the initial misdiagnosis of MODY10—based on an incidental INS variant—and enabled appropriate genetic counseling and discontinuation of unnecessary insulin therapy. CGM emerges as a valuable ancillary tool for distinguishing MODY2 from MODY10 and for reassuring patients that pharmacological treatment is not required.

## Introduction

Maturity-onset diabetes of the young (MODY) comprises a heterogeneous group of autosomal dominant monogenic diabetes disorders characterized by early onset, non-autoimmune β-cell dysfunction, and marked clinical heterogeneity (1). Accurate molecular diagnosis is paramount, as different MODY subtypes require fundamentally distinct management strategies—ranging from lifestyle modification alone to specific pharmacological interventions or insulin therapy (2, 3). Despite advances in genetic testing, MODY remains underdiagnosed globally, with an estimated 80% of patients misclassified as type 1 or type 2 diabetes, leading to years of inappropriate treatment (3, 4).

Among MODY subtypes, MODY2 and MODY10 represent two clinically distinct entities with opposing therapeutic approaches, yet they share sufficient phenotypic overlap to create diagnostic confusion. MODY2, caused by heterozygous inactivating mutations in the glucokinase gene (GCK), is one of the most common MODY subtypes globally, accounting for up to 80% of cases in Spain (5) and 55.4% in a recent Chinese pediatric cohort (6). GCK mutations reset the glucose homeostasis threshold (typically 5.4–8.3 mmol/L), leading to mild, stable fasting hyperglycemia with minimal postprandial excursions and rare microvascular complications (7, 8). Crucially, patients with MODY2 rarely benefit from pharmacological intervention, as exogenous insulin or oral hypoglycemic agents do not significantly alter the genetically determined glucose set point (3, 9). In contrast, MODY10, caused by mutations in the insulin gene (INS), typically presents with progressive β-cell dysfunction due to misfolded proinsulin accumulation in the endoplasmic reticulum, leading to ER stress and apoptosis via dominant-negative effects (10, 11). These patients often require pharmacological treatment or insulin therapy, particularly when diagnosed in childhood or presenting with higher HbA1c levels (12, 13). The clinical differentiation between MODY2 and MODY10 is therefore critical: while MODY2 patients exhibit stable, mild hyperglycemia with preserved C-peptide, MODY10 patients typically show progressive glucose deterioration, glycemic instability, and may develop insulin dependence (14, 15).

Diagnostic confusion between MODY subtypes remains common, particularly when genetic testing reveals incidental variants or when clinical features overlap. Copy number variations (CNVs), including large deletions, are increasingly recognized as causes of MODY2 but may be missed by standard sequencing approaches (1, 16). With the widespread application of next-generation sequencing (NGS), incidental variants are frequently identified; however, their pathogenicity requires rigorous validation through segregation analysis, population frequency assessment, and formal classification under ACMG/AMP criteria (12). Failure to do so can lead to misdiagnosis and years of unnecessary treatment, as illustrated by the pedigree reported here.

Continuous glucose monitoring (CGM) has emerged as a promising ancillary tool in monogenic diabetes diagnostics. Previous studies have shown that CGM-derived metrics, particularly the coefficient of variation (CV) and glucose distribution patterns, can distinguish GCK-MODY from well-controlled type 2 diabetes with an area under the curve (AUC) of 0.875 (17, 18). The characteristic “plateau-like” stable hyperglycemia pattern of MODY2 contrasts sharply with the “peak-and-trough” glycemic instability seen in MODY10, where mutant proinsulin misfolding causes ER stress and β-cell dysfunction, leading to increasingly unstable insulin secretion (15, 19). CGM provides dynamic, real-time insights into glycemic physiology that static laboratory tests cannot capture, offering a non-invasive window into the functional consequences of underlying genetic defects (19, 20).

## Case presentation

### Patient information and clinical evaluation

The proband was a 4-year-10-month-old Chinese male referred to the Endocrine and Genetic Metabolism Clinic at Beijing Children’s Hospital, Capital Medical University, in June 2025. He was identified during routine screening due to a family history of diabetes. The child was asymptomatic, with no polyuria, polydipsia, weight loss, or recurrent infections. Birth weight was 3200 g (full-term normal delivery, appropriate for gestational age).

The family pedigree spans five generations with autosomal dominant transmission of diabetes (**Figure 1**; **Table 1**). Generation I comprises three deceased individuals: the central male ancestor (filled symbol, diabetic) and two female partners (both unfilled, non-diabetic). Generation II includes offspring from both partnerships. From the first partnership, six siblings are shown (five males and one female), among whom diabetes is suspected in five of six individuals; however, detailed clinical and genetic data are unavailable. From the second partnership, one daughter (filled circle, deceased, diabetic) and her non-diabetic partner (unfilled square, deceased) form the lineage leading to the proband. Generation III includes the maternal grandmother (filled circle, carrier of the heterozygous GCK deletion [+/-], with a 20-year history of diabetes treated with insulin and metformin) and the maternal grandfather (unfilled square, carrier of the INS NM_000207.3:c.4G>A variant [-/+], aged 70 years with normal glucose metabolism [fasting glucose 5.0 mmol/L, HbA1c 5.2%]). An additional branch shows one non-diabetic female (unfilled circle) and one diabetic male (filled square), with one diabetic daughter (filled circle) in Generation IV.Generation IV includes the proband’s mother (filled circle, carrier of both variants [+/+], aged 42 years, diagnosed with gestational diabetes at age 32 years and treated with detemir insulin for ten years, maintaining HbA1c around 6.5% with minimal insulin requirements [0.1–0.2 U/kg/day]) and the proband’s father (unfilled square, wild-type [-/-], non-diabetic). Despite insulin therapy, the mother’s fasting glucose ranged from 5.4–8.3 mmol/L, and she reported no hypoglycemic episodes, admitting to irregular insulin administration.Generation V includes two male siblings: the proband (arrow, filled square, +/+, 4 years 10 months old, with fasting plasma glucose 6.61 mmol/L and HbA1c 6.5%) and his elder brother (filled square, +/+, 10 years old, with elevated fasting glucose 6.3–6.5 mmol/L and HbA1c 6.3–6.5%, currently untreated).Although the pedigree spans five generations, the present study focused on six individuals across Generations III to V for whom detailed clinical, genetic, and continuous glucose monitoring data were available.

**Figure 1:**
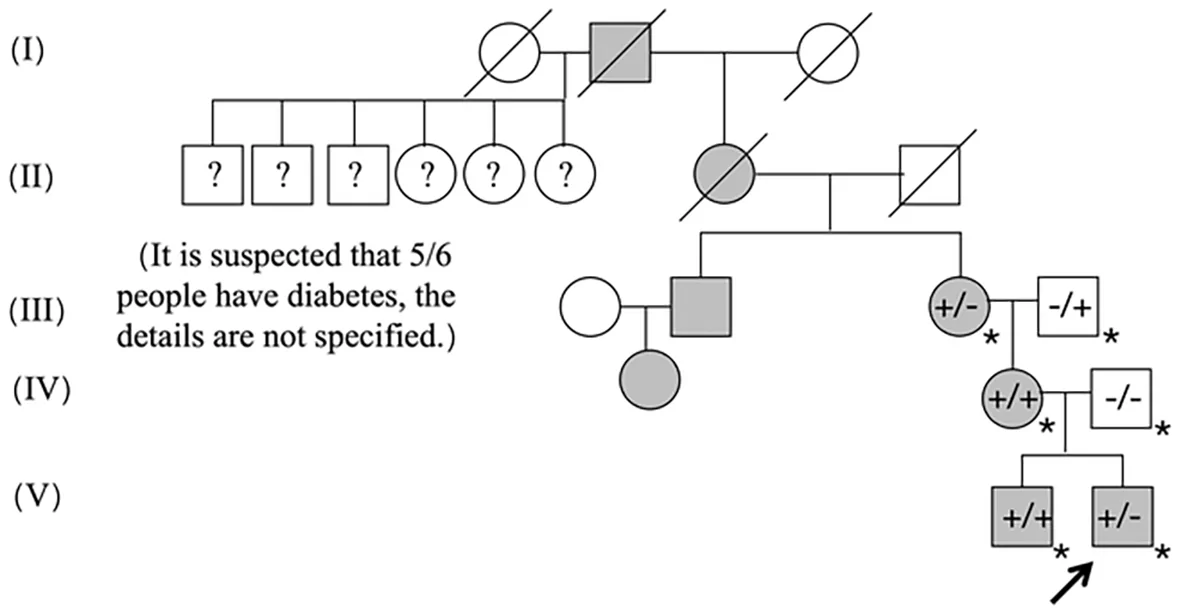
Pedigree of the Chinese family showing autosomal dominant inheritance of diabetes across five generations and genetic segregation analysis. Arrow indicates the proband (4-year-10-month-old boy, individual V-1). Asterisks () mark family members who underwent genetic testing. Black filled symbols denote clinically diagnosed diabetes; slashed symbols indicate deceased individuals; question marks indicate family members with unknown clinical status. Gray filled symbols with slash indicate deceased individuals with diabetes. Genotypes are indicated as follows: “+/-” (heterozygous for the GCK deletion), “-/+” (heterozygous for the INS c.4G>A variant), “+/+” (carrier of both variants), and “-/-” (wild-type for both).

**Table 1:** Clinical characteristics of the proband and affected family members.

Characteristic	Proband (4y10m)	Mother (42y)	Elder brother (10y)	Maternal grandmother	Maternal grandfather
Age at diagnosis	4y10m	32y (GDM)	10y	60+y	N/A
BMI (kg/m²)	15.8	22.1	18.5	24.0	23.5
Fasting glucose (mmol/L)	6.61	5.4–8.3	6.3–6.5	7.0–8.5	5.0
HbA1c (%)	6.5	6.5	6.3–6.5	7.2	5.2
Treatment	Lifestyle	Detemir insulin (discontinued)	Lifestyle	Insulin + Metformin	None
GCK deletion	Positive	Positive	Positive	Positive	Negative
INS c.4G>A	Negative	Positive	Positive	Negative	Positive

Physical examination of the proband revealed normal growth parameters (height 110 cm, 50th percentile; weight 18 kg, 50th percentile; BMI 15.8 kg/m²). No acanthosis nigricans, lipodystrophy, or dysmorphic features were observed. Pubertal staging was Tanner stage I. Blood pressure was normal (95/60 mmHg).

### Biochemical and metabolic phenotyping

Laboratory investigations revealed fasting plasma glucose of 6.61 mmol/L and HbA1c of 6.5%. Diabetes autoantibodies (anti-GAD, anti-IA-2, anti-ZnT8, and IAA) were all negative. Fasting C-peptide was 0.77 ng/mL (normal range 0.8–4.0 ng/mL), indicating preserved β-cell function. Renal and hepatic functions were normal. Urinalysis was negative for glucose and ketones.

The proband’s mother underwent reassessment upon presentation of her son. Despite ten years of insulin therapy, her HbA1c remained stable at 6.5%. Her fasting C-peptide was detectable at 0.6 ng/mL, suggesting retained endogenous insulin secretion inconsistent with typical autoimmune type 1 diabetes or severe MODY10.

### Genetic analysis

Initial whole-exome sequencing (WES) performed at an external institution identified a heterozygous INS variant (NM_000207.3:c.4G>A; p.Ala2Thr; rs535989053) in the mother, leading to a preliminary diagnosis of MODY10. The proband and his elder brother were subsequently tested: the proband did not carry the variant.

However, the genetic findings were inconsistent with the clinical phenotype. Segregation analysis revealed that the INS variant was inherited from the maternal grandfather, who had normal glucose tolerance (fasting glucose 5.0 mmol/L, HbA1c 5.2%). Conversely, the diabetic maternal grandmother did not carry the variant. Furthermore, the proband tested negative for the INS variant. This violation of autosomal dominant inheritance patterns necessitated re-evaluation.

Trio-WES with CNV analysis was performed. Sequence alignment revealed significantly reduced read depth in GCK exon 1, suggesting a heterozygous deletion (**Figure 2A**). Multiplex ligation-dependent probe amplification (MLPA) confirmed a heterozygous deletion encompassing GCK exon 1 in the proband, mother, and elder brother, while the father showed normal dosage (**Figure 2B**).

**Figure 2:**
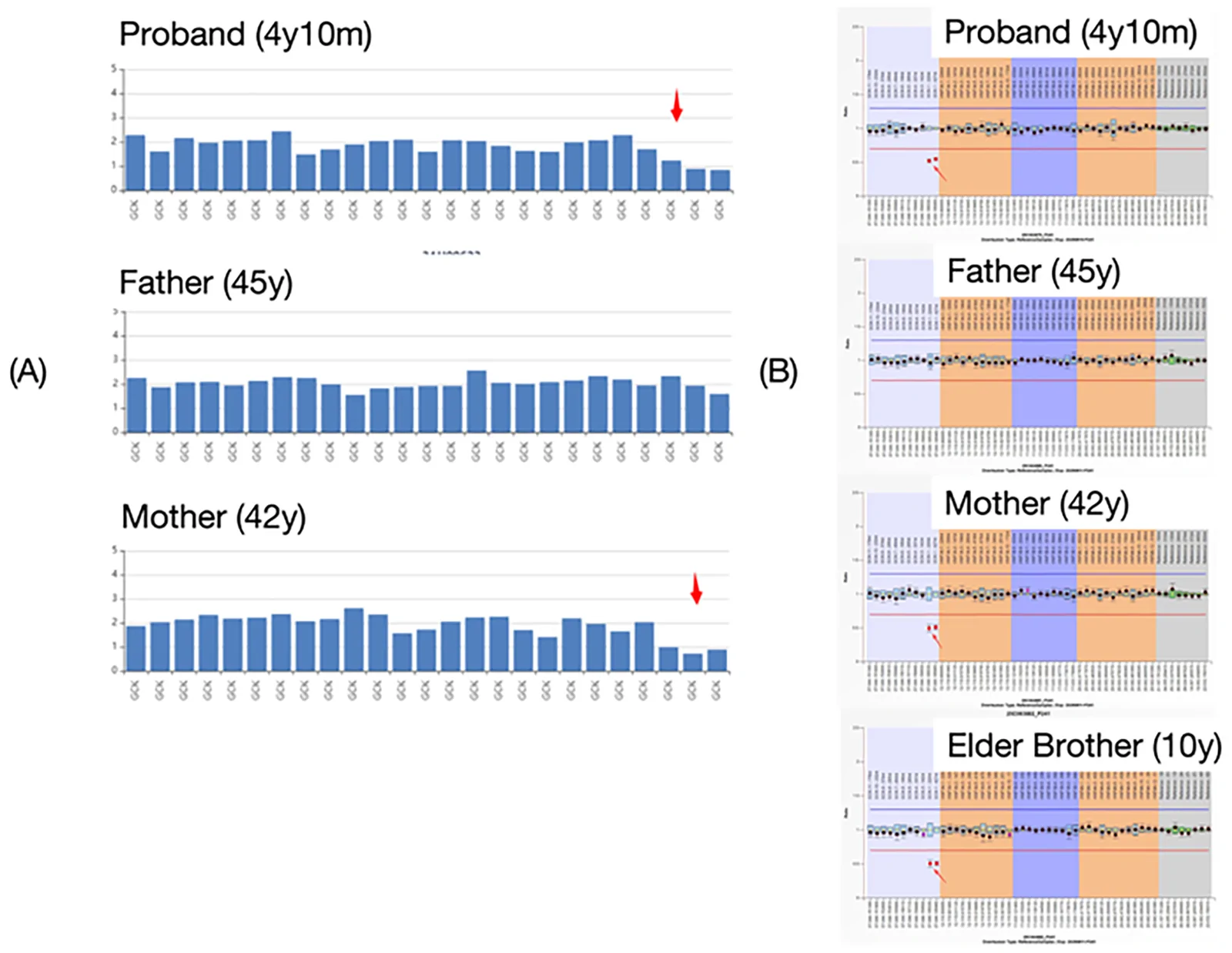
MLPA-based confirmation of GCK exon 1 heterozygous deletion. **(A)** Initial CNV analysis of trio-WES data (proband, mother, and father) revealed reduced read depth in GCK exon 1 in the proband and mother compared to flanking exons, suggestive of a heterozygous deletion (indicated by red arrows). The father showed normal copy number across all exons. **(B)** Due to the inherent limitations of NGS in detecting single-exon heterozygous deletions, multiplex ligation-dependent probe amplification (MLPA) was performed for confirmation.

Whole-genome sequencing (WGS) precisely mapped the deletion breakpoints to chr7:44, 228, 224-44, 229, 394 (GRCh37/hg19), spanning 1, 171 bp (**Figures 3A, B**). This region includes the β-cell promoter, exon 1, and part of intron 1 of the GCK gene. Sanger sequencing across the breakpoint confirmed the deletion, showing a heterozygous junction fragment in affected individuals (**Figure 3C**). This specific deletion has not been previously reported in the literature or variant databases.

**Figure 3:**
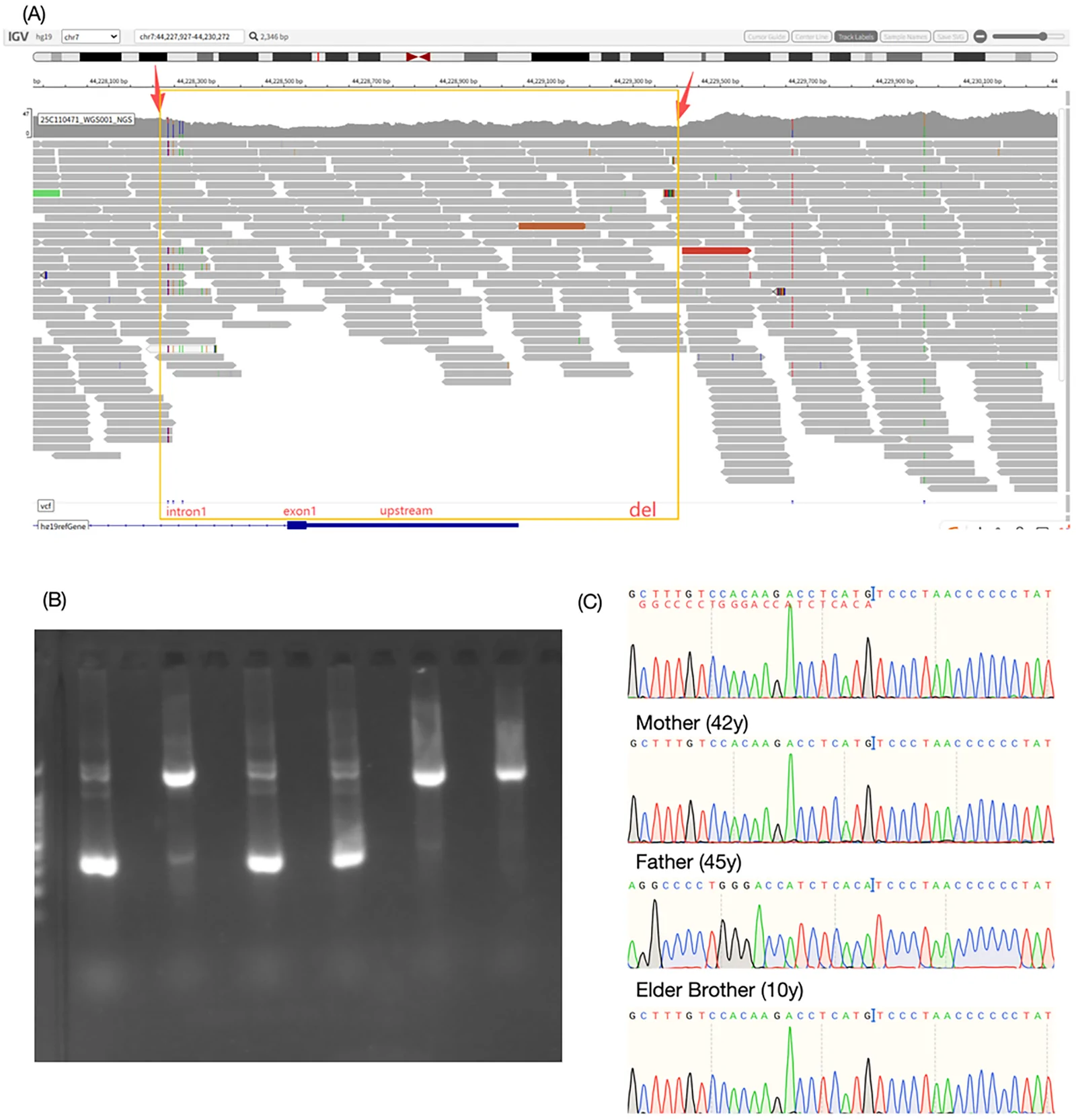
WGS-based breakpoint mapping, PCR validation, and Sanger sequencing confirmation of the GCK heterozygous deletion. **(A)** Integrative Genomics Viewer (IGV) screenshot showing WGS read alignment across the GCK gene region (chr7:44, 227, 927-44, 230, 272). The yellow box highlights the 1, 171 bp deletion (chr7:44228224-44229394, indicated by red arrows), encompassing the promoter region, exon 1, and partial intron 1. Significantly reduced read depth in this region compared to flanking sequences is consistent with a heterozygous deletion. The gene structure diagram below shows the deleted region (del) spanning exon 1 and upstream regulatory sequences. **(B)** Agarose gel electrophoresis (2%) of PCR products amplified across the deletion breakpoint using primers flanking chr7:44228224-44229394. Lane 1: Mother (42y); Lane 2: Father (45y); Lane 3: Elder Brother (10y); Lane 4: Proband (4y10m); Lane 5-6: Negative control. Affected individuals (Proband, Mother, and Brother) show two distinct bands: the upper band (~500 bp) represents the wild-type allele, and the lower band (~380 bp) represents the deleted allele (1171 bp deletion). Unaffected individuals (Father) display only the single wild-type band (~500 bp). **(C)** Representative Sanger sequencing chromatograms confirming the heterozygous breakpoint junction (chr7:44228224-44229394). The vertical dashed line indicates the breakpoint position. Affected individuals (Proband, Mother, and Elder Brother) exhibit overlapping double peaks at the junction, demonstrating the presence of both wild-type and deleted sequences (heterozygous deletion: +/-). In contrast, the Father shows clean single peaks throughout, consistent with the wild-type genotype (-/-). The double-peak pattern confirms the 1, 171 bp deletion and demonstrates co-segregation with the diabetes phenotype across three generations. .

Segregation analysis confirmed that the GCK deletion was inherited from the grandmother (who carries the deletion), while the grandfather does not carry the deletion. The proband, who carries the deletion but not the INS variant, exhibits elevated blood glucose levels, confirming the pathogenicity of the GCK deletion. The grandfather, who carries the INS variant, has normal blood glucose levels, demonstrating that this variant is non-pathogenic in this family (**Figure 1**; **Table 1**).

After the molecular diagnosis was confirmed, insulin was discontinued for two weeks in the proband’s mother while she wore a continuous glucose monitoring (CGM) device; blood glucose levels did not deteriorate. The proband and the elder brother received lifestyle interventions alone and both wore CGM devices. We compared and analyzed the CGM data of all three individuals (**Table 2**; **Figure 4**).

**Table 2:** CGM-derived metrics in the proband, elder brother, and mother.

Parameter	Proband	Mother	Elder Brother
Mean glucose (MG), mmol/L	7.27	7.02	7.74
Coefficient of variation (CV), %	14.35	18.52 (↑)	13.41
Time in range (TIR, 3.9-10.0 mmol/L), %	98.46	97.36	96.17
Time below range (TBR, <3.9 mmol/L), %	0.00	0.83	0.00
Time above range (TAR, >10.0 mmol/L), %	1.54	1.81	3.83
Glucose risk index (GRI)	1.23	3.62	3.06
CONGA, mmol/L	0.97	1.23	1.03
ADRR	1.07	1.32	0.95
Monitoring duration, days	7	14	9
Number of data points	583	1.326	784
Hypoglycemic events (n)	0	[1]	0
Lowest glucose recorded, mmol/L	—	2.4	—

TIR, time in range; TBR, time below range; TAR, time above range; GRI, glucose risk index;CONGA, continuous overlapping net glycemic action; ADRR, average daily risk range.[] indicates the only individual with hypoglycemic events.

**Figure 4:**
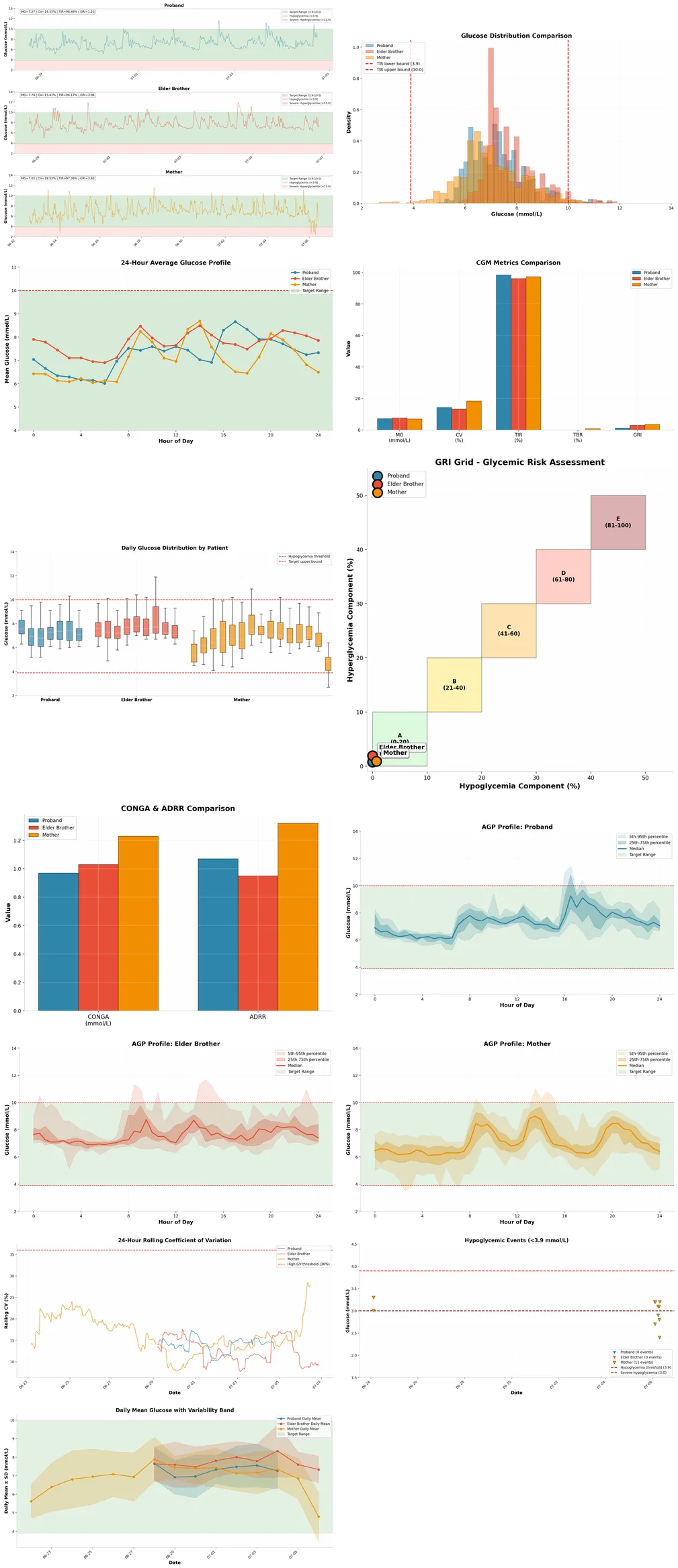
**(A)** CGM Time-Series Traces **(A)** Continuous glucose monitoring (CGM) time-series traces from three family members. The upper, middle, and lower panels show the proband (MODY2 alone), elder brother (GCK deletion + INS variant), and mother (GCK deletion + INS variant), respectively. Green shaded zone: target range (3.9–10.0 mmol/L); red shaded zone: hypoglycemia (<3.9 mmol/L); dark red shaded zone: severe hyperglycemia (>13.9 mmol/L). Proband (Upper panel, blue): Demonstrates the classic “plateau-like” stable mild hyperglycemia pattern of MODY2, with glucose fluctuating within a narrow band of 6.0–9.0 mmol/L. Postprandial peaks are modest (<10 mmol/L), and nocturnal glucose remains stable without hypoglycemic episodes. MG 7.27 mmol/L, CV 14.35%, TIR 98.46%, GRI 1.23. This pattern reflects the elevated glucose-sensing threshold characteristic of GCK-MODY, where β-cell function remains stable despite the reset set-point (17). Elder Brother (Middle panel, red): Shows a stable pattern similar to the proband, with modest postprandial excursions. MG 7.74 mmol/L, CV 13.41% (the lowest of the three), TIR 96.17%, GRI 3.06. The absence of marked glycemic deterioration argues against a modifying effect of the INS variant. Mother (Lower panel, orange): Exhibits a “peak-and-trough” pattern, with postprandial spikes to 10–11.6 mmol/L and nocturnal hypoglycemic dips (nadir 2.4 mmol/L). MG 7.02 mmol/L, CV 18.52%, TIR 97.36%, TBR 0.83%, GRI 3.62. These hypoglycemic events occurred during and shortly after discontinuation of ten years of insulin therapy and are consistent with iatrogenic hypoglycemia in a patient with MODY2 rather than a primary effect of the INS variant. **(B)** Glucose Distribution Histograms **(B)** The Proband (blue) shows a narrow, symmetric unimodal distribution centered at ~7.3 mmol/L with minimal tail extension into hyperglycemic ranges, reflecting the “static offset” characteristic of MODY2. The Elder Brother (red) shows a similarly narrow distribution. The Mother (orange) displays a wider distribution with leftward extension into the hypoglycemic range (<3.9 mmol/L), attributable to recent insulin withdrawal. **(C)** 24-Hour Average Glucose Profile **Figure 3**. The Proband (blue) maintains the flattest diurnal profile with postprandial increments <2 mmol/L and nocturnal stability at 6–7 mmol/L. The Elder Brother (red) shows modest dawn phenomenon and postprandial peaks. The Mother (orange) demonstrates the most pronounced postprandial excursions, with mean glucose frequently exceeding 10 mmol/L during daytime hours and early morning hypoglycemic nadirs during the insulin discontinuation period. **(D)** CGM Metrics Comparison Bar Chart **(D)** Side-by-side comparison of five key CGM metrics: Mean Glucose (MG), Coefficient of Variation (CV), Time in Range (TIR), Time Below Range (TBR), and Glycemia Risk Index (GRI). All three individuals are presented. Notably, the Elder Brother (carrier of both variants) has the lowest CV (13.41%), which does not support a worsening effect of the INS variant on glycemic variability. **(E)** Daily Glucose Distribution Boxplots **(E)** Box-and-whisker plots of daily glucose distributions arranged by patient. Each box represents one monitoring day. Red dashed lines indicate hypogycemia threshold (3.9 mmol/L) and target upper bound (10.0 mmol/L). The Proband and Elder Brother show consistently narrow interquartile ranges without outliers beyond the target range. The Mother shows the widest day-to-day variability, with lower outliers <3.9 mmol/L on multiple dates (most prominent on July 6), coinciding with insulin discontinuation. **(F)** GRI Grid — Glycemic Risk Assessment **(F)** Glycemia Risk Index (GRI) grid plot. X-axis: hypoglycemia component (%); Y-axis: hyperglycemia component (%). The Proband (blue) is closest to the origin (GRI = 1.23). The Elder Brother (red) is displaced toward higher hyperglycemia components (GRI = 3.06). The Mother (orange) is displaced toward both hypoglycemia and hyperglycemia components (GRI = 3.62), reflecting iatrogenic hypoglycemia from recent insulin cessation. **(G)** CONGA and ADRR Comparison **(G)** Comparison of Continuous Overlapping Net Glycemic Action (CONGA, 60-minute interval) and Average Daily Risk Range (ADRR). The Mother shows the highest CONGA (1.23 mmol/L), reflecting increased short-term fluctuations during the insulin withdrawal period. **(H)** AGP-Style Percentile Profile (Proband) **(H)** Ambulatory Glucose Profile (AGP)-style percentile curves for the Proband (MODY2 alone). The 5th–95th percentile band is tight (~5.5–9.5 mmol/L). Nocturnal hours (00:00–06:00) show the narrowest percentile band, with median glucose stable at 6.5–7.5 mmol/L. **(I)** AGP-Style Percentile Profile (Elder Brother) **(I)** AGP-style percentile curves for the Elder Brother. The 5th–95th percentile band is similar to the proband (~5.0–10.5 mmol/L), with modest postprandial expansion. Nocturnal percentile bands are preserved. **(J)** AGP-Style Percentile Profile (Mother) **(J)** AGP-style percentile curves for the Mother. The 5th–95th percentile band is widest (~4.0–11.5 mmol/L), showing early morning hours (02:00–08:00) with the 5th percentile dropping below 4 mmol/L, reflecting nocturnal hypoglycemia during insulin withdrawal. **(K)** 24-Hour Rolling Coefficient of Variation **(K)** Time-series plot of rolling CV calculated over 24-hour windows. The Proband (blue) maintains a stable rolling CV around 14–16%. The Elder Brother (red) shows stable rolling CV around 12–16%. The Mother (orange) exhibits the most variable rolling CV (14–22%), with spikes coinciding with hypoglycemic events during the insulin discontinuation period. **(L)** Hypoglycemic Events Detail **(L)** Detailed scatter plot of all hypoglycemic events (<3.9 mmol/L). The Proband and Elder Brother show zero events. The Mother (orange) shows 11 hypoglycemic events, clustering in the early morning hours (02:00–08:00, 7 events), consistent with iatrogenic nocturnal hypoglycemia during insulin withdrawal. Three events were severe (<3.0 mmol/L), with a nadir of 2.4 mmol/L (July 6, 2025 at 07:01). **(M)** Daily Mean Glucose with Variability Band **(M)** Daily mean glucose (solid lines) ± 1 standard deviation (shaded bands) plotted against calendar date. The Proband and Elder Brother show stable daily means with narrow SD bands. The Mother shows pronounced day-to-day variation, with a marked mean glucose dip on July 6 coinciding with the cluster of hypoglycemic events during insulin discontinuation.

### Ethics statement

This study protocol was approved by the Ethics Committee of Beijing Children’s Hospital, Capital Medical University [(Approval No.: IEC-C-006-A04-V.08 (2026)-E-068-R)]. All procedures adhered to the Declaration of Helsinki. Written informed consent was obtained from the proband’s legal guardians and all adult participants for genetic testing, CGM monitoring, and scientific publication. For minors, assent was obtained where age-appropriate, and parental consent was provided for all procedures.

### Study design and participants

We conducted a detailed clinical and genetic evaluation of a Chinese pedigree spanning three generations. The proband was a 4-year-10-month-old boy referred to the Endocrine and Genetic Metabolism Clinic at Beijing Children’s Hospital in June 2025 due to a family history of diabetes. Family members underwent systematic phenotyping including biochemical profiling, oral glucose tolerance testing (OGTT), and metabolic assessment. Clinical characteristics were documented in a standardized case report form.

### Genetic analysis

Initial Whole-Exome Sequencing (WES): WES was performed at an external institution on the proband’s mother, followed by targeted Sanger sequencing of the INS gene in the proband and elder brother.

Trio-WES with CNV Analysis: Upon identification of inconsistent segregation patterns, trio-WES (proband, mother, father) with integrated CNV analysis was performed. Sequence alignment and read depth analysis were conducted using standard bioinformatics pipelines to detect heterozygous deletions.

Multiplex Ligation-Dependent Probe Amplification (MLPA): MLPA was performed using the SALSA MLPA Probemix P241-C1 GCK kit (MRC-Holland, Amsterdam, The Netherlands) to confirm the heterozygous deletion encompassing GCK exon 1. Dosage ratios <0.75 or >1.25 were considered indicative of a deletion or duplication, respectively.

Whole-Genome Sequencing (WGS): WGS was performed to precisely map the deletion breakpoints. PCR amplification across the breakpoint region was conducted using primers flanking chr7:44, 228, 224-44, 229, 394 (GRCh37/hg19), followed by Sanger sequencing of the junction fragment.

Segregation Analysis: Sanger sequencing of the GCK deletion breakpoint and the INS c.4G>A variant was performed in all available family members to confirm genotype-phenotype correlations.

ACMG/AMP Variant Classification: Both variants were classified according to the ACMG/AMP guidelines and the ClinGen Monogenic Diabetes Expert Panel specifications for GCK and INS (21, 22). Population frequency data were retrieved from gnomAD (v2.1.1 and v3.1.2) and the 1000 Genomes Project.

### Biochemical and metabolic phenotyping

Fasting plasma glucose, HbA1c, C-peptide, and diabetes autoantibodies (anti-GAD, anti-IA-2, anti-ZnT8, and IAA) were measured using standard laboratory methods. OGTT was performed according to the World Health Organization protocol. Renal and hepatic functions were assessed by routine biochemical assays.

### Continuous glucose monitoring

After molecular diagnosis was confirmed, the proband’s mother discontinued insulin therapy for two weeks and wore a Flash Continuous Glucose Monitoring System (Freestyle Libre H, Abbott Diabetes Care Ltd). The proband and elder brother, managed with lifestyle intervention alone, also wore Freestyle Libre H devices. Monitoring duration was 7 days for the proband, 9 days for the elder brother, and 14 days for the mother. Data were exported and analyzed at 15-minute intervals for consistency.

CGM metrics were calculated as follows:

Mean Glucose (MG): Average of all glucose values during the monitoring period.

Coefficient of Variation (CV): (Standard deviation/MG) × 100%.

Time in Range (TIR): Percentage of time with glucose 3.9–10.0 mmol/L.

Time Below Range (TBR): Percentage of time with glucose <3.9 mmol/L.

Time Above Range (TAR): Percentage of time with glucose >10.0 mmol/L.

Glycemia Risk Index (GRI): Calculated according to the standardized formula combining hypoglycemia and hyperglycemia components.

Continuous Overlapping Net Glycemic Action (CONGA): Calculated at 60-minute intervals.

Average Daily Risk Range (ADRR): Mean of daily risk scores.

### Statistical analysis

Descriptive statistics were used to summarize clinical and CGM data. Continuous variables are presented as mean ± standard deviation (SD) or median (interquartile range), as appropriate. Categorical variables are presented as frequencies and percentages.

For CGM metric comparisons among the three family members (isolated MODY2 proband, elder brother carrying both variants, and mother carrying both variants), descriptive comparisons were performed. Due to the family-based design and limited sample size (n=3), formal inferential statistics were not applied; instead, we report absolute values and interpret findings with appropriate caution.

Correlation between CGM metrics and clinical parameters was assessed using Pearson’s correlation coefficient. Statistical analyses were performed using R version 4.3.1 (R Foundation for Statistical Computing, Vienna, Austria) or SPSS version 26.0 (IBM Corp., Armonk, NY, USA). A two-sided p-value <0.05 was considered statistically significant.

### Data availability

The datasets generated and analyzed during the current study are available from the corresponding author on reasonable request. Genetic data are subject to controlled access to protect participant privacy, in accordance with the approved ethics protocol. The GCK deletion has been submitted to the ClinVar database (accession number pending).

## Discussion

This study presents a Chinese pedigree in which a large 1, 171 bp GCK deletion (encompassing the β-cell promoter, exon 1, and part of intron 1) was identified as the true causative variant for MODY2, correcting an initial misdiagnosis of MODY10 based on an incidental INS variant. We demonstrate that this GCK deletion alone causes mild, stable fasting hyperglycemia consistent with the classic MODY2 phenotype. Furthermore, our CGM data reveal that continuous glucose monitoring-derived metrics—particularly the coefficient of variation (CV), Glycemia Risk Index (GRI), and hypoglycemic event profiles—can serve as non-invasive, real-time biomarkers to discriminate isolated MODY2 from MODY10, thereby guiding genetic testing strategies and therapeutic decisions.

### ACMG/AMP classification and the incidental INS variant

A central finding of this study is that the INS NM_000207.3:c.4G>A (p.Ala2Thr) variant is not the cause of diabetes in this pedigree. Under ACMG/AMP criteria, this variant meets evidence for benign classification. First, it is present in population databases at an allele frequency incompatible with a fully penetrant dominant MODY10 allele: the gnomAD East Asian allele frequency is approximately 1.2 × 10^-^³ in genomes and 4.3 × 10^-4^ in exomes, with a frequency of ~1 × 10^-^³ in 1000 Genomes East Asians (23). For a rare autosomal dominant monogenic disorder, this frequency far exceeds the threshold for BS1 (benign stand-alone). Second, the variant did not co-segregate with diabetes: the 70-year-old maternal grandfather, who carries the variant, has had normal glucose tolerance throughout his life (fasting glucose 5.0 mmol/L, HbA1c 5.2%), providing strong BP2 evidence (observed in a healthy adult). Third, the proband, who has diabetes, does not carry the variant. Collectively, these data classify the INS c.4G>A variant as benign (BS1, BP2).

In contrast, the GCK deletion is classified as pathogenic under ACMG/AMP criteria using the ClinGen Monogenic Diabetes Expert Panel specifications. It meets PVS1 (null variant—deletion of exon 1 and the promoter predicts loss of function), PM2 (absent from controls in the gnomAD and 1000 Genomes databases), and PP1 (co-segregation with diabetes in three generations). The precise 1, 171 bp deletion coordinates (chr7:44, 228, 224-44, 229, 394) remove the entire pancreatic transcript exon 1 (chr7:44, 228, 508-44, 229, 022), approximately 372 bp of upstream β-cell promoter, and approximately 284 bp of intron 1, exactly as determined by WGS and validated by Sanger sequencing across the junction.

### Tissue-specific promoter loss and mechanistic insight

GCK is transcribed from two tissue-specific promoters. The deletion identified in this pedigree removes the β-cell promoter and exon 1 while leaving the hepatic promoter intact. This predicts selective loss of the pancreatic glucokinase isoform with hepatic glucokinase unaffected, providing a clear mechanistic explanation for the pure β-cell phenotype of mild fasting hyperglycemia without hepatic involvement. This is a real point of interest distinguishing this allele from the coding variants that make up most of the GCK literature, and it underscores why patients with this deletion present with the classic MODY2 phenotype: an elevated glucose-sensing threshold in pancreatic β-cells while hepatic glucose metabolism remains normal.

### CGM phenotyping and comparison of all three genotypes

Our CGM data provide direct evidence that the GCK deletion alone produces the characteristic stable hyperglycemia of MODY2, and that the presence of the benign INS variant does not markedly alter this pattern. The proband (isolated MODY2) exhibited a “plateau-like” stable mild hyperglycemia pattern: MG 7.27 mmol/L, CV 14.35%, TIR 98.46%, and GRI 1.23, consistent with the established CGM signature of GCK-MODY described in the literature (17, 18). Previous studies have shown that GCK-MODY patients display higher fasting glucose set-points with relatively low postprandial glucose excursions, reflecting the elevated glucose-sensing threshold due to GCK haploinsufficiency (8, 24). Our proband’s CGM profile precisely mirrors this pattern, with glucose remaining within a narrow band (6.0–9.0 mmol/L) and gentle postprandial rises rarely exceeding 10 mmol/L.

Importantly, the elder brother, who carries both the GCK deletion and the INS variant, showed a CGM profile that was not markedly worse than the proband’s: his CV was 13.41% (the lowest of the three), and his mean glucose was 7.74 mmol/L. This directly contradicts the hypothesis that the co-occurrence of the INS variant worsens glycemic variability. The mother, who also carries both variants, exhibited a CV of 18.52% and 11 hypoglycemic events (nadir 2.4 mmol/L); however, these events occurred during her insulin taper and shortly after discontinuation, reflecting iatrogenic hypoglycemia from years of unnecessary exogenous insulin therapy in a patient with MODY2, rather than a primary effect of the INS variant. Her GRI (3.62) was elevated primarily due to these hypoglycemic events. When all three individuals are compared, there is no consistent gradient supporting a modifying effect of the INS variant (**Table 2**). Instead, the data support the conclusion that the GCK deletion alone determines the phenotype, while the INS variant is clinically silent.

### Novelty in the context of previous GCK deletions

While this specific 1, 171 bp promoter/exon 1 deletion is novel, GCK deletions causing MODY2 are not without precedent. Ellard et al. (2007) provided the foundational description of partial and whole gene deletions of GCK and HNF1A in MODY, identifying an exon 2 deletion and establishing that CNVs account for approximately 3% of mutations in these genes (25). More recently, Yu et al. (2024) reported a Chinese pedigree with a GCK exon 8–10 deletion detected by WES-based CNV analysis and confirmed by MLPA, with the same diagnostic message emphasizing the importance of copy number analysis when sequencing is negative (26). Our case extends this literature by describing the first reported deletion specifically affecting the β-cell promoter and exon 1, highlighting the mechanistic consequence of selective pancreatic isoform loss.

### Clinical implications and CGM-guided management

Our findings have direct clinical implications for the management of MODY patients. First, the extremely low GRI (1.23) and absence of hypoglycemia in the proband confirm that isolated MODY2 does not require pharmacological intervention, and CGM can serve to reassure patients and prevent unnecessary treatment (3, 8, 9). This aligns with expert consensus that GCK-MODY patients typically do not require any glucose-lowering medication, as pharmacological agents cannot override the genetically determined glucose set-point (3, 27).

Second, the misdiagnosis of MODY2 as insulin-dependent diabetes led to a decade of unnecessary insulin therapy in the mother, exemplifying the harm of over-treatment in GCK-MODY. Studies demonstrate that discontinuing pharmacological therapy in GCK-MODY patients results in minimal change in HbA1c (average change −0.06%, 95% CI −0.27% to 0.15%), confirming that lifestyle modification alone is sufficient for most patients without insulin resistance (8, 9). Indeed, approximately 31% of MODY2 patients exhibit insulin resistance, and only this subset might benefit from metformin therapy; in the absence of insulin resistance, conventional hypoglycemic agents are ineffective (3, 8).

For pregnancy management in GCK-MODY, fetal growth depends on the fetal genotype rather than maternal glycemic control; insulin therapy is indicated only when fetal abdominal circumference exceeds the 75th percentile, indicating the fetus is unaffected by the mutation and at risk for macrosomia (7, 28). The mother in this pedigree, diagnosed with “gestational diabetes” at age 32 and treated with insulin for ten years, likely had undiagnosed GCK-MODY during pregnancy. Her stable HbA1c (6.5%) despite minimal insulin doses (0.1–0.2 U/kg/day) and irregular administration was a classic clue to MODY2, where exogenous insulin cannot override the genetically determined glucose set-point (7, 9).

### The critical role of CNV analysis in monogenic diabetes diagnostics

The initial misclassification of this pedigree as MODY10, based solely on an incidental heterozygous INS variant, underscores a pervasive diagnostic pitfall in the era of NGS. With widespread high-throughput sequencing, incidental variants are frequently identified; however, their pathogenicity requires rigorous validation through segregation analysis, population frequency assessment, and formal ACMG/AMP classification (12, 29). This case highlights a crucial limitation of conventional WES: standard pipelines may have insufficient coverage or analytical sensitivity for detecting CNVs, particularly intragenic deletions affecting single exons (1, 16). In MODY2, over 600 different mutations have been documented, including large deletions that demand specific detection strategies such as MLPA, WGS-based CNV analysis, or targeted array comparative genomic hybridization (aCGH) (1, 16).

We strongly recommend that clinicians and geneticists incorporate CNV analysis into the diagnostic algorithm for suspected monogenic diabetes, particularly when WES returns negative results despite compelling clinical suspicion (stable mild fasting hyperglycemia, multigenerational diabetes with autosomal dominant inheritance, and preserved C-peptide). The identification of the 1, 171 bp GCK deletion in this pedigree—missed by initial WES but detected through trio-WES with CNV analysis and confirmed by MLPA—demonstrates that comprehensive genetic evaluation must extend beyond point mutation detection to include gene dosage analysis.

### Limitations

This study has several limitations. First, the sample size is inherently limited by the family-based design, with only three individuals undergoing CGM comparison. Our findings therefore require validation in larger cohorts. Second, we did not perform functional experiments (e.g., *in vitro* enzyme assays for the GCK deletion or proinsulin folding studies for the INS variant) to directly validate the pathogenic mechanisms. The pathogenicity of the GCK deletion was inferred from co-segregation analysis and the proband’s phenotype, while the benign nature of the INS c.4G>A variant was inferred from segregation and population frequency data rather than molecular functional data. Third, our CGM monitoring periods varied among family members (7, 9, and 14 days), which may introduce variability in metric reliability, although the mother’s 14-day period exceeds the minimum recommendation for CGM data interpretation (20). Fourth, the generalizability of our CGM-based discrimination strategy to other MODY subtypes or ethnic populations requires further investigation, as existing CGM phenotyping data are predominantly derived from European cohorts (4).

## Conclusions and future directions

In conclusion, this case illustrates the critical diagnostic journey from a misdiagnosis of MODY10 to the correct identification of MODY2, emphasizing that accurate molecular diagnosis prevents unnecessary insulin therapy and enables appropriate genetic counseling. The identification of a 1, 171 bp GCK deletion underscores the importance of CNV analysis and segregation studies in monogenic diabetes workups, particularly when WES returns negative results or reveals incidental variants that violate expected inheritance patterns. The benign classification of the INS c.4G>A variant serves as a cautionary reminder that not all rare variants in MODY genes are pathogenic, and rigorous variant interpretation—including family segregation, population frequency assessment, and formal ACMG/AMP classification—is essential. Finally, CGM emerges as a promising ancillary tool for discriminating MODY2 from MODY10 and for reassuring patients that pharmacological treatment is unnecessary, offering dynamic, real-time insights into glycemic physiology that static laboratory tests cannot provide.

We recommend that clinicians encountering diagnostic confusion in MODY evaluation should: (1) incorporate CNV analysis (MLPA or WGS-based) into the genetic workup when standard sequencing is negative; (2) perform rigorous segregation analysis for all identified variants, particularly those that appear incidental or violate autosomal dominant inheritance; (3) apply formal ACMG/AMP classification using disease-specific expert panel criteria before assigning pathogenicity; (4) consider CGM as a complementary diagnostic tool to characterize glycemic patterns and reassure patients that MODY2 does not require pharmacological intervention; and (5) maintain awareness that incidental findings in MODY genes can lead to years of unnecessary treatment if not rigorously validated.

Future studies should validate CGM-based discrimination algorithms in larger, multi-ethnic MODY cohorts, and functional studies should elucidate the precise molecular consequences of promoter-specific GCK deletions on pancreatic β-cell glucose sensing.

## Data Availability

The datasets generated and analyzed during the current study are available from the corresponding author on reasonable request. Genetic data are subject to controlled access to protect participant privacy, in accordance with the approved ethics protocol. The GCK deletion has been submitted to the ClinVar database (accession number pending).
